# Tumor P-Glycoprotein Correlates with Efficacy of PF-3758309 in *in vitro* and *in vivo* Models of Colorectal Cancer

**DOI:** 10.3389/fphar.2013.00022

**Published:** 2013-03-22

**Authors:** Erica Lynn Bradshaw-Pierce, Todd M. Pitts, Aik-Choon Tan, Kelly McPhillips, Mark West, Daniel L. Gustafson, Charles Halsey, Leslie Nguyen, Nathan V. Lee, Julie L. C. Kan, Brion William Murray, S. Gail Eckhardt

**Affiliations:** ^1^Pfizer Global Research and DevelopmentLa Jolla, CA, USA; ^2^Department of Pharmaceutical Sciences, University of Colorado DenverAurora, CO, USA; ^3^University of Colorado Cancer Center, University of Colorado DenverAurora, CO, USA; ^4^Division of Medical Oncology, University of Colorado DenverAurora, CO, USA; ^5^Department of Clinical Science, Colorado State UniversityFort Collins, CO, USA

**Keywords:** P-glycoprotein, PF-3758309, colorectal cancer, intrinsic resistance, mouse xenografts

## Abstract

P-glycoprotein (P-gp), a member of the ATP-binding cassette transporter family, is overexpressed in a number of different cancers and some studies show that P-gp overexpression can be correlated to poor prognosis or therapeutic resistance. Here we sought to elucidate if PF-3758309 (PF-309), a novel p-21 activated kinase inhibitor, efficacy was influenced by tumor P-gp. Based on *in vitro* proliferation data, a panel of colorectal cancer cell lines were ranked as sensitive or resistant and *ABCB1* (P-gp) expression was evaluated by microarray for these cell lines. P-gp expression was determined by western blot and activity determined by rhodamine efflux assay. Knock down of P-gp and pharmacologic inhibition of P-gp to restore PF-309 activity was performed *in vitro*. PF-309 activity was evaluated *in vivo* in cell line xenograft models and in primary patient derived tumor xenografts (PDTX). Mice were treated with 25 mg/kg PF-309 orally, twice daily. On the last day of treatment, tumor and plasma were collected for PF-309 analysis. Here we show that *ABCB1* gene expression correlates with resistance to PF-309 treatment *in vitro* and the expression and activity of P-gp was verified in a panel of resistant cells. Furthermore, inhibition of P-gp increased the sensitivity of resistant cells, resulting in a 4–100-fold decrease in the IC50s. Eleven cell line xenografts and 12 PDTX models were treated with PF-309. From the cell line xenografts, we found a significant correlation between *ABCB1* gene expression profiles and tumor response. We evaluated tumor and plasma concentrations for eight tumor models (three cell line xenografts and five PDTX models) and a significant correlation was found between tumor concentration and response. Additionally, we show that tumor concentration is approximately fourfold lower in tumors that express P-gp, verified by western blot. Our *in vitro* and *in vivo* data strongly suggests that PF-309 efficacy is influenced by the expression of tumor P-gp.

## Introduction

Multi-drug resistance (MDR) proteins and their expression or overexpression has been correlated to poor prognosis, therapeutic resistance, and relapse in various cancer types (Sinicrope et al., 1994; Fardel et al., 1996; Sekine et al., 2009; Zajchowski et al., 2012). P-glycoprotein (P-gp), encoded by *ABCB1* (also known as *MDR1*), is the most well-studied energy-dependent transmembrane efflux protein, with a wide range of structurally diverse substrates (Linn and Giaccone, 1995; Fardel et al., 1996; Gottesman et al., 2002; Lee et al., 2010). Breast cancer resistance protein (BCRP), encoded by *ABCG2*, is another drug efflux transporter that has been shown to play a role in resistance to various cancer therapeutics. P-gp and BCRP are expressed in some normal tissues, to serve as protective barriers (i.e., in brain, placenta, testes) or for detoxification (i.e., in liver, kidney intestine), and is expressed at high levels in numerous tumor types (Gottesman et al., 2002; Leonard et al., 2003; Lee et al., 2010). Typically, tumors derived from epithelial tissues that normally express P-gp such as colorectal, hepatocellular, and renal cancer, frequently express high levels of P-gp (Linn and Giaccone, 1995; Gottesman et al., 2002; Leonard et al., 2003).

In oncology, resistance to therapy is a critical issue and can be due to molecular or genetic alterations that affect sensitivity or impair drug delivery. The overexpression of MDR proteins has been correlated with both *de novo* (Sinicrope et al., 1994; Sekine et al., 2009) and acquired (Zajchowski et al., 2012) resistance to therapeutics in cancer. The presence or upregulation of MDR proteins has been demonstrated in a variety of cancer types and has been shown to contribute to reduced intracellular concentration of substrates (McGrath and Center, 1988). Extensive preclinical and clinical studies have focused on MDR proteins in cancer resistance to chemotherapeutics (Bellamy et al., 1988; McGrath and Center, 1988; Jang et al., 2001a,b; Leonard et al., 2003; Murray et al., 2012); however, the role of MDR proteins in resistance to the newer signal transduction inhibitors is not well-understood.

PF-3758309 (PF-309) is a novel pyrrolopyrazole inhibitor of p-21 activated kinase (PAK) that has demonstrated low nanomolar activity in a range of cancer types (Murray et al., 2010). The PAK family of proteins, PAKs 1–3 (group I) and PAKs 4–6 (group II), play a role in the regulation of cell motility, survival, angiogenesis, and proliferation (Kumar et al., 2006; Eswaran et al., 2009; Molli et al., 2009). Additionally, PAKs are altered in a variety of cancers, and increased migratory potential, anchorage-independent growth, oncogenic transformation, and metastasis has been tied to overexpression of PAKs (Eswaran et al., 2009; Murray et al., 2010); making PAKs an attractive therapeutic target (Kumar et al., 2006; Eswaran et al., 2009; Molli et al., 2009).

The current focus in oncology is to design and develop agents that target signal transduction pathways. Often times these agents are only beneficial to a select population of patients and identification of biomarkers for the prediction of response has gained significant attention (Banck and Grothey, 2009; Pitts et al., 2010; Prenen et al., 2010; Siddiqui and Piperdi, 2010; Tentler et al., 2010; Heakal et al., 2011; Shaw et al., 2011). However, for compounds that are MDR transporter substrates, it may be beneficial to also: (1) determine how strongly the compound efficacy is altered by the presence of transporters; and (2) use transporter expression/activity information in conjunction with molecular classifiers to select patients with the highest likelihood to benefit from the therapy. In the studies presented here we show that PF-309 is a substrate for the MDR transporters P-gp and BCRP, and we sought to determine if PF-309 activity is influenced by the presence of tumor transporters in *in vitro* and *in vivo* models of colorectal cancer (CRC). CRC is a cancer type in which P-gp overexpression is commonly observed (Linn and Giaccone, 1995) and PAKs have been shown to be overexpressed and play a role in disease progression (Carter et al., 2004; Kumar et al., 2006; Tabusa et al., 2013).

## Materials and Methods

### Chemicals and reagents

PF-3758309 (molecular weight 490.63) was provided by Pfizer, Inc (La Jolla, CA, USA). PF-309, CP-100356, and Ko143 stock solutions for *in vitro* experiments were prepared in DMSO (Fisher Scientific, Fairlawn, NJ, USA). All other materials used were purchased from either Fisher Scientific (Fairlawn, NJ, USA) or Sigma (SLouis, MO, USA) unless specified.

### Cells and culture conditions

All colorectal, breast, and lung cancer cell lines, except GEO, were all obtained from ATCC (Manassas, VA, USA). GEO cells were a generous gift from Dr. Fortunato Ciardiello (Cattedra di Oncologia Medica, Dipartimento Medico-Chirurgico di Internistica Clinica e Sperimentale “F Magrassi e A Lanzara,” Seconda Università degli Studi di Napoli, Naples). All cells, except GEO, were grown in RPMI medium supplemented with 10% fetal bovine serum, 1% non-essential amino acids, 1% penicillin/streptomycin. GEO cells were grown in DMEM/F12 supplemented with 10% fetal bovine serum, 1% non-essential amino acids, and 1% penicillin/streptomycin. All cells were maintained at 37°C in a humidified incubator with 5% CO_2_. Cells were routinely screened for the presence of mycoplasma (MycoAlert, Cambrex Bio Science, Baltimore, MD, USA) and were tested and authenticated in the University of Colorado Cancer Center DNA Sequencing and Analysis Core. CRC cell line DNA was tested using the Profiler Plus Kit (Applied Biosystems, Foster City, CA, USA) and compared to that from the ATCC. All *in vitro* drug treatments were conducted with the use of complete growth media.

### MDCKII-LE, MDR1-MDCK, and BCRP-MDCKII-LE transwell assays

Permeability and transporter substrate assays were performed as previously published (Feng et al., 2008; Callegari et al., 2011; Di et al., 2011). Permeability assays were conducted in MDCKII-LE cells, which were generated by isolation of a subpopulation of cells with low expression of endogenous P-gp (Feng et al., 2008; Callegari et al., 2011; Di et al., 2011). The use of MDCKII-LE cells are used to measure the passive permeability of compounds in place of MDCK wild-type or Caco-2 cells since both MDCK wild-type and Caco-2 cells are known to have some level of endogenous transporter expression which can affect the permeability measurement. P-gp efflux was determined in MDR1-MDCK cells and BCRP efflux was measured in BCRP-MDCKII-LE cells.

### Cells proliferation assays

Cytotoxic/proliferation effects were determined using the sulforhodamine B (SRB) assay (Skehan et al., 1990) or by resazurin. Briefly, cells in logarithmic growth phase were transferred to 96 well flat bottom plates with lids. One-hundred microliter cell suspensions containing 1500–5000 viable cells were plated into each well and incubated overnight prior to exposure with increasing concentrations of PF-309 for 3–5 days, dependent upon cell type. Media was removed and cells were fixed with cold 10% trichloroacetic acid for 30 min at 4°C. Cells were then washed with water and stained with 0.4% SRB (Fisher Sci., Pittsburgh, PA, USA) for 30 min at room temperature, washed again with 1% acetic acid, followed by stain solubilization with 10 mM tris at room temperature. The plate was then read on a plate reader (Biotek Synergy 2, Winooski, VT, USA) set at an absorbance wavelength of 565 nm. Cell proliferation curves were derived from the raw absorbance (OD) data. For P-gp and BCRP inhibition assays, cells were incubated with increasing concentrations of PF-309 in the presence or absence of 0.625 μM CP-100356 or 0.8 μM Ko143 for 72 h then assayed by SRB or CellTiter-Glo^®^ (Promega).

### Western blot

Cells were seeded into 6-well plates 24 h prior to harvest. Cells were scraped into RIPA buffer containing protease inhibitors, EDTA, NaF, and sodium orthovanadate. Total protein was determined (BioRad D_c_ Protein Assay, BioRad, Hercules, CA, USA) and 50 μg of total protein was loaded onto a 4–12% gel, electrophoresed and transferred to Immobilon-P (Millipore, Bedford, MA, USA). Membranes were blocked for 1 h at room temperature with 5% non-fat dry milk in TBS containing tween-20 (0.1%) then incubated overnight at 4°C with antibodies to P-gp (1:250 dilution, Santa Cruz) or actin (1:1000 dilution, Cell Signaling, Beverly, MA, USA). Following primary antibody incubation, blots were washed in TBS-Tween (0.1%), then incubated with the appropriate secondary antibody at 1:15,000 (LI-COR, Lincoln, NE, USA) for 1 h at room temperature. Following three additional washes, blots were developed using the Odyssey Infrared Imaging System (LI-COR Biosciences).

### Rhodamine uptake assay

Rhodamine uptake/efflux assays were conducted similarly to a previously published report (Lee et al., 1994). Cells were seeded into 6-well plates 24 h prior to the efflux assay. Rhodamine (3 μM) or rhodamine + CP-100356 (1 μM) was added to plated cells. After 1 h, rhodamine containing media was removed and fresh media or media + CP-100356 was added to cells for 1 h of efflux. Cells were harvested, washed and flow cytometry performed to measure the fluorescence intensity per 5000 cells.

### shRNA knockdown

The pGIPZ Pgp-specific shRNA expression cassettes, along with control shRNA plasmids including the original pGIPZ vector were purchased from Open Biosystems (Birmingham, AL, USA). Pgp-specific shRNA plasmids were co-transfected with packaging plasmids into HEK293T to produce lentiviral particles, according to manufactures instructions. Stable clones were generated by transducing CRC cell lines with lentiviral particles containing Pgp-specific shRNAs. Seventy-two hours after transduction, the cells were placed under selection with 2.5 μg/mL of puromycin, splitting 1:5 when the cells reached confluency. Multiple clones from the same transfection were pooled and grown under puromycin selection. Successful knockdown of specific genes and gene products was confirmed by semi-quantitative RT-PCR and immunoblotting with specific antibodies. Each experiment was conducted in triplicate.

### Animals

Female 5–6-week-old athymic nude mice were purchased from Harlan Sprague Dawley. Animals were housed (three to five per cage) in polycarbonate cages and kept on a 12 h light/dark cycle. Animals were allowed to acclimate for at least 7 days before any handling. Food and water were given *ad libitum*. All studies were conducted at the University of Colorado Anschutz Medical Campus in accordance with the National Institutes of Health guidelines for the care and use of laboratory animals, and animals were housed in a facility accredited by the American Association for Accreditation of Laboratory Animal Care.

### Xenograft studies

#### Cell line xenografts

For cell line xenografts, CRC cells were harvested in exponential phase growth and resuspended in a 1:1 mixture of serum-free RPMI 1640 and Matrigel (BD Biosciences). Five to ten million cells per mouse were injected subcutaneously into the flank using a 23-gauge needle. Mice were monitored daily for signs of toxicity and were weighed twice weekly. Tumor size was evaluated twice per week by caliper measurements using the following formula: tumor volume = length × width^2^ × 0.52. When tumors reached 200–300 mm^3^ mice were randomized into two groups with at least 10 tumors per group. Mice were then treated for 14–28 days (until control tumor volume reach ∼1500 mm^3^) with either vehicle control (0.5% methylcellulose), or PF-3758309 (25 mg/kg) twice daily by oral gavage.

#### Patient derived tumor xenografts

For patient tumor xenografts, surgical specimens of patients undergoing either removal of a primary or metastatic tumor at the University of Colorado Hospital are implanted subcutaneously into five mice for each patient with a 10-G trochar. After a subsequent growth and passage in mice, tumors are excised, and expanded into cohorts for treatment (*n* = 6–15 per group). Mice were treated with PF-309 or vehicle (0.5% methylcellulose) for 22–56 days (until control tumor volume reach ∼1500 mm^3^) twice daily (BID) by oral gavage (PO). Monitoring of mice and measurements of tumors were conducted as described above.

### GENE EXPRESSION PROFILES

#### CRC cell lines gene expression profiles

Cells were plated at 2 × 10^6^ in 6-well plates 24 h prior to harvest. After 24–72 h cells were rinsed twice with PBS, and RNA was prepared using a RNeasy Plus mini kit (Qiagen, Valencia, CA, USA). RNA stabilization, isolation, and microarray sample labeling were carried out using standard methods for reverse transcription and one round of *in vitro* transcription. Total RNA isolated from CRC cell lines was hybridized on Affymetrix U133 Plus 2.0 microarrays. The sample preparation and processing procedure was performed as described in the Affymetrix GeneChip^®^ Expression Analysis Manual (Affymetrix Inc., Santa Clara, CA, USA). In addition, CRC cell line gene expression profiles were obtained from the GlaxoSmithKline (GSK) genomic profiling data via the NCI cancer Bioinformatics Grid (caBIG^®^) website^1^. These data were also profiled using Affymetrix U133 Plus 2.0 gene arrays in triplicates. To integrate the data generated from our lab and GSK, absolute intensity signals from the microarray gene expression profiles were extracted and probe sets representing the same gene were collapsed based on maximum values. Next, the gene expression levels were converted to a rank-based matrix and standardized (mean = 0, standard deviation = 1) for each microarray. Using this pre-processing method, the same cell lines from different data sets were clustered based on their gene expression profiles. Data analyses were performed on this rank-based matrix.

#### CRC cell line xenograft gene expression profiles

Total RNA isolated from CRC cell line xenografts was hybridized on Affymetrix HuGene 1.0 ST microarrays. The sample preparation and processing procedure was performed as described in the Affymetrix GeneChip^®^ Expression Analysis Manual (Affymetrix Inc., Santa Clara, CA, USA). Gene expression was normalized by the RMA method using Affymetrix Power Tools.

#### Epithelial and mesenchymal genetic signature

For determining the epithelial to mesenchymal signature, epithelial (E; *CLDN2*, *CDH1*, *RAB25*, *CLDN3*, and *CDH17*), and mesenchymal (M) genes (*CALD1*, *VIM*, *ANK2*, and *ZEB1*) derived from the PAK inhibitor (see companion article: (Pitts et al., 2013). The expressions of these genes were ranked normalized within cell line and then Z-normalization was performed on this rank-based matrix. An average value is computed for the E-genes and M-genes. Gene expression of the *ABCB1* was also obtained by ranked normalized. To visualize the heatmap, we used the matrix2png software (Pavlidis and Noble, 2003) where rows and columns represent genes and cell lines, respectively. Red and green colors represent high and low expression, respectively.

### Plasma and tumor concentrations

Plasma and tumor samples were collected 1 h post treatment at the end of therapeutic studies or 6 h post treatment for P-gp tumor concentration studies. PF-309 was measured in mouse plasma and tumor tissue using a validated LC/MS/MS assay carried out by the University of Colorado Cancer Center Pharmacology Shared Resource. Positive ion electrospray ionization (ESI) mass spectra were obtained with a 3200 QTrap triple quadrupole mass spectrometer (Applied Biosystems Inc., Foster City, CA, USA) with a turboionspray source interfaced to an Agilent 1200 Series binary HPLC system (Agilent Technologies Inc., Santa Clara, CA, USA), and a HTC-PAL auto-sampler (LEAP Technologies, Carrboro, NC, USA). Samples were chromatographed with a Waters Sunfire 2.5 μm C18 column (4.6 × 50 mm; Waters Corp., Milford, MA, USA) with a Phenomenex C18 filter frit guard cartridge (Phenomenex, Torrance, CA, USA). The LC elution was done with a gradient starting at 10% Acetonitrile with 0.1% formic acid:90% 0.1% formic acid held for 1.5 min followed by a 1-min linear gradient to 98% Acetonitrile with 0.1% formic acid: 2% 0.1% formic acid at a flow rate of 750 μl/min and a sample volume of 50 μl. The total analysis time was 5.5 min.

The mass spectrometer settings were: source temperature, 600°C, ion spray voltage, 4500 V, source gas, 50, curtain gas (CUR), 10, collision gas (CAD), low, and needle position 3. Transition specific parameters used were 491.2 → 284.2/491.2 → 148.3/372.2 → 176.1: declustering potential (DP), 46.32/46.54/56.86 V, exit potential (EP), 6.45/3.23/4.08 V, collision cell entrance potential (CEP), 25.92/27.80/20.56 V, collision energy (CE), 40.00/53.10/32.75 V, collision cell exit potential (CXP), 0.42/1.47/2.72 V. Samples were quantified by the internal standard reference method in the MRM mode by monitoring for PF-3758309 transitions of *m/z* 491.2 → 284.2 and 491.2 → 148.3, and for the internal standard (trazodone) of *m/z* 372.2 → 176.1. Unknown, standard and quality control samples were extracted from mouse plasma or tumor homogenates (100 mg/ml) by the sequential addition of 5 μl 500 ng/ml internal standard (trazodone), 250 μl acetonitrile:methanol (3:1) to 50 μl plasma/tumor homogenate. Samples were then vortexed for 5 min followed by centrifugation for 10 min at 17,000 rpm. 200 μl of supernatant was then transferred to a microcentrifuge tube followed by the addition of 400 μl 0.1% formic acid followed by vortexing (5 min) and centrifugation (5 min at 13,300 rpm) and the supernatant collected (600 μl) and transferred to auto-sampler vials. The average accuracy and precision based on analysis of quality control samples was 93.1 and 98.7% for plasma samples (range: 1–2500 ng/mL) and 91.9 and 98.2% for tumor (range: 5–5000 ng/mL).

### Data analysis

Statistical analysis was performed using GraphPad Prism Software version 4.02 (La Jolla, CA, USA). For comparisons of two groups an un-paired *t*-test was performed. For correlations, a Spearman correlation was used. *P* values < 0.05 were considered statistically significant.

## Results

### PF-3758309 is a substrate for P-glycoprotein and breast cancer resistance protein

MDCKII-LE, MDR1-MDCK, and BCRP-MDCKII-LE transwell assays were performed to determine the permeability and transporter efflux of PF-309 *in vitro* (Table 1). The permeability of PF-309 was determined to be 0.9 × 10^−6^ cm/s in MDCKII-LE cells (mean of two replicates is presented). As previously described (Feng et al., 2008), in MDCKII-LE cells a compound with a donor to receiver (A → B) flux of <5 × 10^−6^ cm/s is classified as having low permeability. Compounds with A → B flux of 5–15 × 10^−6^ cm/s are classified as moderately permeable and compounds >15 × 10^−6^cm/s are considered to be highly permeable. Therefore, PF-309 is classified as a poorly permeable compound by the MDCKII-LE assay.

**Table 1:** **PF-3758309 permeability and efflux**.

	A → B (cm/s)	B → A (cm/s)	Efflux ratio
MDCK-MDR1 efflux	0.33 ± 0.06	11.5 ± 1.5	34.9 ± 7.9
BCRP-MDCKII-LE efflux	;1.4 ± 0.1	10.5 ± 1.3	;7.6 ± 1.1
MDCKII-LE permeability assay	;0.9 × 10^−6^ cm/s		

MDR1-MDCK and BCRP-MDCKII-LE transwell assays measure the effect of P-gp and BCRP, respectively, on transcellular flux. The efflux ratio, ratio of the B → A flux to the A → B flux, of PF-309 was measured to be 34.9 ± 7.9 in the MDR1-MDCK assay, indicating it is a strong substrate for P-gp. PF-309 was also determined to be substrate for BCRP as the efflux ratio was measured to be 7.6 ± 1.1. As a reference, compounds with efflux ratios >2 are considered to be substrates (Callegari et al., 2011).

### P-glycoprotein expression correlates with *in vitro* activity

**Figure 1:**
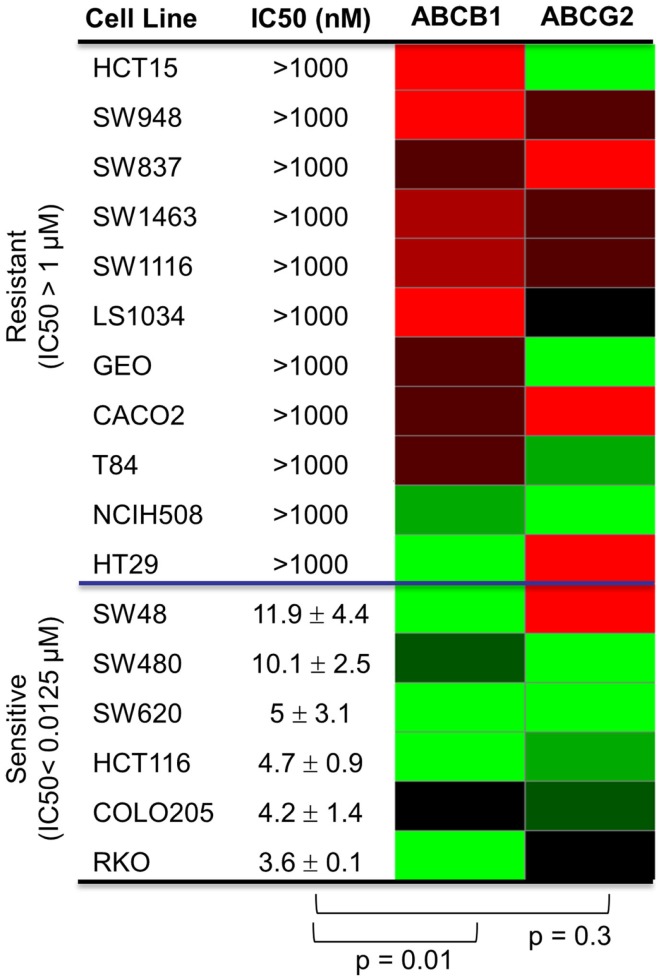
**Correlation of *ABCB1* and *ABCG2* expression and resistance in a panel of CRC cell lines**. Anti-proliferative effects of PF-309 was determined in CRC cell lines treated with various concentrations for 72 h by SRB assay. Cell line IC50 values were calculated and values below 12.5 nM were classified as sensitive and cell lines with an IC50 greater than 1 μM were classified as resistant (∼100-fold difference in activity). A heatmap for *ABCB1* and *ABCG2* expression in the panel of lines was generated (red represents high expression and green represents low). Spearman correlation of *ABCB1* relative expression and IC50 values showed a significant correlation (*p* = 0.01) while *ABCG2* relative expression did not correlate with activity (*p* = 0.3).

**Figure 2:**
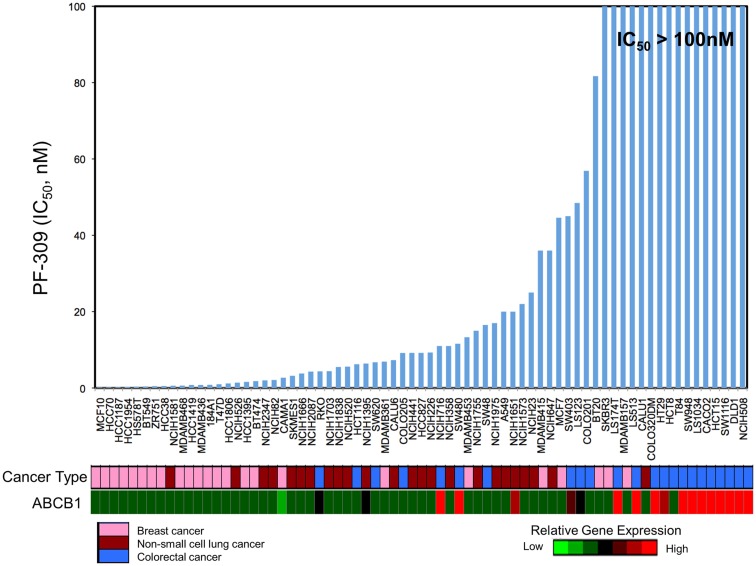
**Correlation of *ABCB1* expression and resistance in an expanded panel of cell lines**. A heatmap for *ABCB1* expression in a panel of CRC, breast, and non-small cell lung cancer cell lines was generated and IC50 values established by resazurin (red represents high expression and blue/black represents low). Again, a significant correlation between *ABCB1* gene expression and IC50 was found (*p* < 0.0001).

Since PF-309 appeared to be a strong P-gp and BCRP substrates, we sought to evaluate if the transporters influenced sensitivity *in vitro*. We evaluated the sensitivity of CRC cell lines to PF-309 treatment in a panel of 27 lines. Cells were exposed to PF-309 at a range of concentrations for 72 h and cell content measured by SRB assay (see Pitts et al., 2013). From the 27 lines tested, we selected lines that had approximately 100-fold difference in IC50 and examined the *ABCB1* and *ABCG2* gene expression profiles (Figure 1). We found a significant correlation between relative *ABCB1* levels in CRC cell lines and their respective IC50s (*p* = 0.01); but, no correlation was found between relative *ABCG2* and IC50s (*p* = 0.3). A similar analysis was conducted in an expanded panel of 71 cell lines to include CRC, breast, and non-small cell lung cancer cell lines (Figure 2). PF-309 activity was evaluated in CRC, breast, and lung cancer lines since PAKs have been shown to be overexpressed in these cancers (Kumar et al., 2006; Ong et al., 2011). Again, a significant correlation between relative *ABCB1* levels and IC50 values was found (*p* < 0.0001).

**Figure 3:**
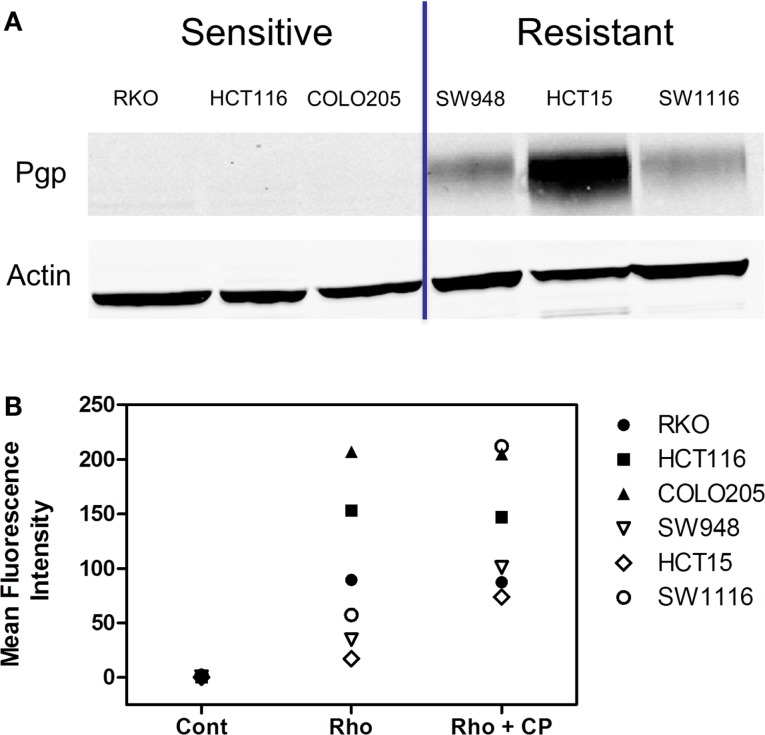
**Expression and activity of P-gp in a panel of CRC cell lines**. **(A)** The presence of P-gp was determined in three sensitive and three resistant cell lines by western blot. Protein expression in this panel of lines corresponded to *ABCB1* expression. **(B)** The activity of P-gp in the same panel of cell lines was verified by rhodamine uptake studies. Rhodamine (Rho) is effluxed by P-gp, so in lines with functional P-gp a lower fluorescence signal is detected. Administration of a P-gp inhibitor, CP-100356 (CP), increases the fluorescence signal in lines with function P-gp but does not affect the signal in lines without P-gp.

**Figure 4:**
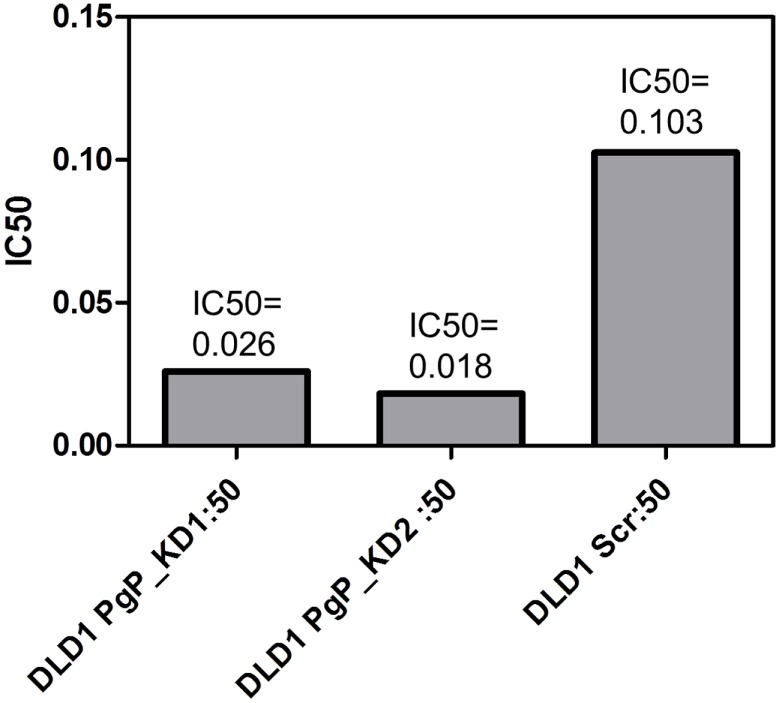
**Knockdown of P-gp by shRNA in DLD-1 cells increased the potency of PF-3758309**. A four to six fold shift in the IC50 was achieved through the knockdown of P-gp.

In a subset of CRC cell lines, western blotting, and rhodamine efflux was used to verify the expression and function of P-gp. Consistent with the gene expression data, the sensitive cell lines, RKO, HCT116, and Colo205 show no P-gp expression and no functional P-gp, and the resistant lines, SW948, HCT15, and SW1116 express functional P-gp (Figures 3A,B). Next we sought to determine if P-gp inhibition could increase the potency of PF-309 in a subset of the CRC cell lines. Proliferation assays were performed with PF-309 in the presence and absence of the P-gp inhibitor CP-100356 (0.625 μM). CP-100356 is a transporter specific (P-gp and BCRP) compound that does not affect metabolism, unlike verapamil and cyclosporine (Kalgutkar et al., 2009). CP-100356 was able to cause a 4–100-fold change in the IC50 of PF-309 in five out of the six CRC cell lines (Table 2). CP-100356 did not directly cause anti-proliferative effects at 0.625 μM and was unable to alter the IC50 of a compound (PF-XXX) that is not a P-gp substrate (IC50 = 440 nM alone compared to 390 nM with CP-100356). In addition to pharmacologic inhibition of P-gp, we determined that shRNA knockdown of P-gp resulted in a four to six fold shift in the IC50 (Figure 4).

**Table 2:** **Inhibition of P-gp increases potency of PF-309**.

Cell line	PF-309 IC50 nM	PF-309 + CP-100356 IC50 nM
DLD-1	278	2.9
Caco-2	300	9.3
HCT15	780	32
LS1034	>1 uM*	796
SW1463	>1 uM*	215
T84	>1 uM*	255

*1 uM was the maximum concentration used. 50% growth inhibition was not achieved at this concentration.

Since, CP-100356 affects both P-gp and BCRP, we also determined if BCRP inhibition alone would alter cell line sensitivity. Proliferation assays were performed with PF-309 in the presence and absence of the BCRP inhibitor Ko143 (0.8 μM). Ko143 was able to enhance the efficacy of PF-309, however only marginally. In HCT15 and Caco-2 cells, the IC50s of PF-309 in the presence of Ko143 was shifted to approximately 500 nM (less than two fold change) and 150 nM (two fold change), respectively.

### *In vivo* efficacy of PF-3758309 correlates with tumor *ABCB1* expression

Human tumor xenograft models were used to evaluate the efficacy of PF-309 *in vivo*. Eleven different CRC cell line tumor xenografts were treated with 25 mg/kg PF-309 twice daily by oral gavage for 14–28 days (until control tumor volumes reached ∼1500 mm^3^). Tumor volumes were measured throughout the study and the % growth inhibition was calculated from the average end of study tumor volumes by:

$$\begin{array}{l} \%\text{ Growth Inhibition}=\\ \;\frac{(\text{average control volume}-\text{average treated volume)}}{\text{average control volume}}\times 100\% \end{array}$$

**Figure 5:**
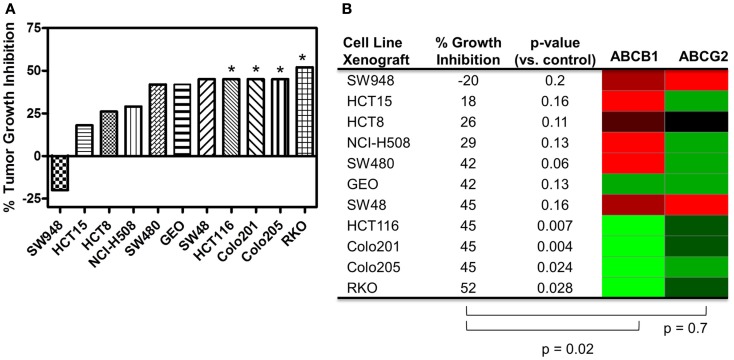
**Correlation of *ABCB1* and *ABCG2* expression and tumor growth inhibition in *in vivo* CRC cell line xenograft models**. **(A)** Percent tumor growth inhibition of 11 CRC cell line xenograft models treated orally with 25 mg/kg PF-309 twice daily. Percent growth inhibition was calculated from end of study tumor volumes in control and treated animals. *p < 0.05 for end of study tumor volumes treated versus control. **(B)** A heatmap for *ABCB1* and *ABCG2* expression in the tumor xenografts was generated from data acquired from baseline tumor samples (red represents high expression and green represents low). Spearman correlation of *ABCB1* relative expression and % growth inhibition showed a significant correlation (*p* = 0.02), while *ABCG2* did not (*p* = 0.7). Tumor models that were unresponsive to treatment (growth inhibition <40%) had high relative levels of *ABCB1*. Conversely, the most responsive tumors had statistically significant differences between controls and treated groups, had average growth inhibition >40% and displayed relative low *ABCB1* levels.

A range of responses was observed with the maximal growth inhibition being 52% (Figure 5). We evaluated the tumor growth inhibition data and *ABCB1* and *ABCG2* gene expression data and found a significant correlation with *ABCB1* (*p* = 0.02), but not with *ABCG2* (*p* = 0.7), reinforcing what was observed *in vitro*. Note that *ABCB1* and *ABCG2* gene expression from baseline (untreated) cell line tumor xenografts was used for this analysis and not the cell line gene expression data.

### *In vivo* efficacy correlates with tumor concentration and P-glycoprotein affects tumor concentration of PF-3758309

**Figure 6:**
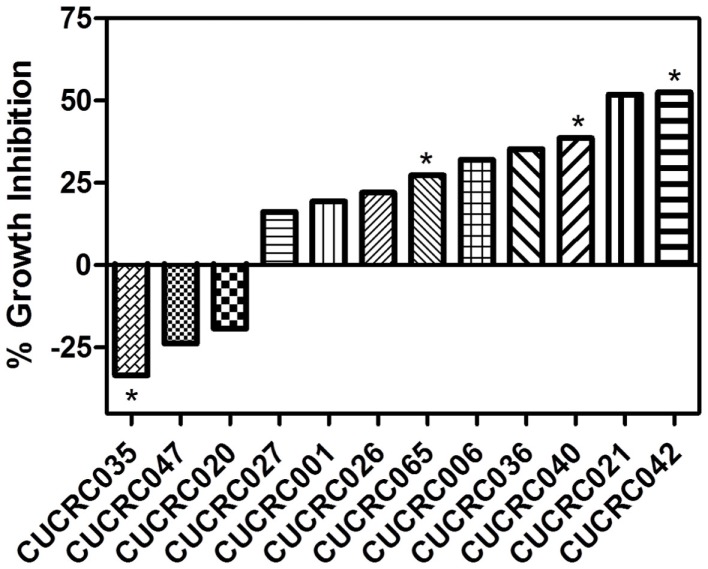
***In vivo* efficacy of PF-309 in CRC PDTX models**. Percent tumor growth inhibition of 12 CRC PDTX models treated orally with 25 mg/kg PF-309 twice daily. Percent growth inhibition was calculated from end of study tumor volumes in control and treated animals. *p < 0.05 for end of study tumor volumes treated versus control. A range of responses was observed similar to what was observed in the cell line xenograft models.

In addition to the cell line xenografts, we also treated 12 different patient derived tumor xenograft (PDTX) CRC models with PF-309 (Figure 6). We were unable to assess how *ABCB1* gene expression correlated with efficacy in these models. The PDTX models are propagated *in vivo*, are not immortalized, and are not grown in *in vitro* culture. Therefore, we have observed some changes in *ABCB1* gene expression in some of our models over time and the direction of the change is not consistent (data not shown). In lieu of analyzing gene expression profiles, we sought to determine if tumor concentration correlated with *in vivo* response. Tumor and plasma sample pairs were collected from animals at the end of study for eight tumor models (three cell line xenografts and five PDTX models) and concentrations of PF-309 were measured. Table 3 shows the paired tumor and plasma concentration data and the calculated tumor to plasma ratio for individual animals (*n* = 2–4 per tumor type) for the different tumor types (each line represents measurements from individual animals). Significant correlations (*p* < 0.003) were found between both tumor concentration and response and tumor to plasma ratios and response (Table 3).

**Table 3:** **End of study tumor and plasma concentrations of PF-309**.

CRC tumor model	% Growth inhibition	Tumor (ng/g)*	Plasma (ng/mL)	Tumor: plasma ratio*
CUCRC042	52	34.8	1.17	29.8
		29.6	4.58	6.46
Colo201	45	140.4	20.9	6.72
		11.1	11.7	0.95
GEO	42	10.2	5.48	1.9
		15.9	54.1	0.3
		22.9	76.6	0.3
CUCRC036	35	65.7	40.6	1.62
		30.7	110	0.28
		47.6	81.3	0.59
CUCRC006	32	3.6	42.4	0.08
		3.4	24.4	0.14
CUCRC026	30	6.4	25.4	0.25
		14.5	114	0.13
		22.7	69.3	0.33
		16.8	184	0.09
HCT15	18	6.8	10.0	0.68
		8.9	13.2	0.67
		8.0	49.0	0.16
CUCRC047	−24	4.4	110	0.04
		7.5	98.7	0.08

*p < 0.003 vs. % growth inhibition.

**Figure 7:**
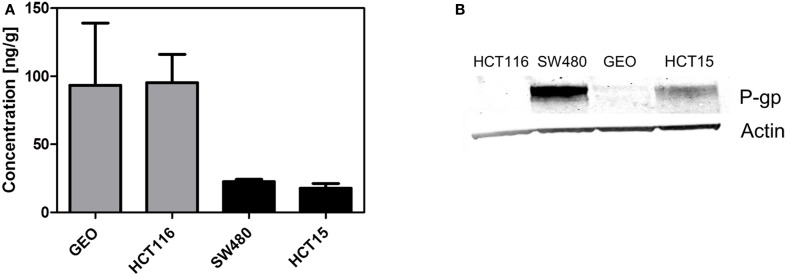
**Decreased tumor concentration of PF-309 corresponds with tumor P-gp expression *in vivo***. **(A)** Tumor concentrations of PF-309 were measured 6 h post treatment of a single 25 mg/kg dose. Higher concentrations of PF-309 were measured in HCT116 and GEO tumors than SW480 and HCT15 tumors. **(B)** Western blots were performed to determine the presence or absence of P-gp in tumors. Corresponding with the higher concentrations observed in HCT116 and GEO tumors, no P-gp was detected. And, in the SW480 and HCT15 tumors where lower concentrations of PF-309 were determined, P-gp was detected.

Finally, we determined if the presence of P-gp affects tumor concentration. We evaluated the tumor concentration in two tumor xenograft lines with high *ABCB1* gene expression (SW480 and HCT15) and two tumor xenografts with low *ABCB1* gene expression (GEO and HCT116). Tumors from animals treated with a single dose of 25 mg/kg PF-309 were collected 6 h post treatment and concentrations analyzed. In tumors with expected high tumor P-gp a ∼four fold lower concentration was observed than the tumors with low *ABCB1* expression (Figure 7A). Tumors were also analyzed by western blot to confirm the presence or absence of P-gp, which agreed with the gene expression data (Figure 7B).

## Discussion

Numerous agents, approved and in development, are substrates for transporters, and it may not be clear yet the role that MDR transporters play in resistance to these therapies. The overall goal of the study described herein was to establish if PF-309 efficacy is influenced by the presence of tumor transporters. We believe that the *in vitro* and *in vivo* data presented here strongly suggests that tumor uptake of PF-309 is a key determinant of efficacy and that tumor P-gp expression affects this.

PF-309 is a strong P-gp substrate and we demonstrated in a panel of cell lines that resistance to PF-309 significantly correlated with *ABCB1* expression. We also showed that PF-309 is a BCRP substrate, but no correlation was found between *ABCG2* relative expression and *in vitro* or *in vivo* efficacy in CRC models. We demonstrated through knockdown and pharmacologic inhibition of P-gp, that activity of PF-309 could be enhanced 4–100-fold in high P-gp expressing lines. *In vivo*, we were able to significantly correlate the efficacy in CRC models to tumor uptake of PF-309 and found a significant correlation between *ABCB1* expression and response in CRC cell line xenograft models. In the cell line xenograft models, we found four models with statistically significant differences in end of study tumor volumes compared with controls that demonstrated >40% average tumor growth inhibition (Figures 5A,B). All four of those tumor lines, HCT116, Colo201, Colo205, and RKO show low *ABCB1* gene expression. Conversely, there were four cell line xenograft models with <40% average tumor growth inhibition, all of which had high *ABCB1* gene expression levels.

Although the *in vitro* data supports a strong influence of P-gp on the activity of PF-309, *in vivo* data is a little less clear. Of the 11 cell line xenografts treated, the presence or absence of P-gp appears to coincide with effect in eight of the models. However, in the remaining three xenograft tumor lines, SW480, GEO, and SW48, the data was inconsistent. In these models we found that similar and reasonable average growth inhibition (42, 42, and 45%) was obtained, although the end of study tumor volumes between treated groups and controls were not statistically significant, but two of the three models had high *ABCB1* gene expression and one did not. As a result, we conducted a study to determine if P-gp was present and if the presence of P-gp altered tumor concentration. For this study we selected two cell line xenografts with results that were not consistent with *ABCB1* expression (SW480 and GEO) and two with consistent results (HCT15, and HCT116). What we found was that P-gp expression agreed with *ABCB1* expression data and that tumors that expressed P-gp had a lower concentration of PF-309 (Figures 7A,B). This data indicates that GEO and SW480 tumors require different concentrations of PF-309 to achieve the same level of activity.

**Figure 8:**
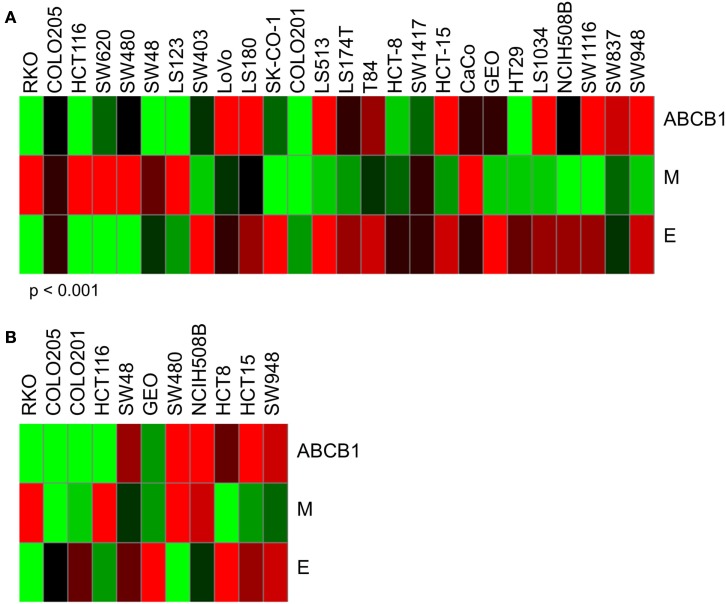
**Heatmaps of *ABCB1* expression and epithelial and mesenchymal genomic signatures**. Determination of epithelial (E) signature was based on expression of *CLDN2*, *CDH1*, *RAB25*, *CLDN3*, and *CDH17*. Mesenchymal (M) signature was based on expression of *CALD1*, *VIM*, *ANK2*, and *ZEB1*. Again, red indicates high expression and green, low. **(A)** *ABCB1*, E, and M expression for *in vitro* CRC cell lines used in these studies. Interestingly, we found a significant correlation between *ABCB1* expression and the epithelial signature (*p* < 0.001). For some of the cell lines that exhibited resistance to PF-309 but did not have high relative *ABCB1* expression, resistance may be attributed to an epithelial genotype. **(B)** *ABCB1*, E, and M expression for the 11 *in vivo* CRC cell line xenografts. No significant correlation between *ABCB1* and E-type was observed here.

Through studies conducted prior to and in conjunction with this work (see companion article (Pitts et al., 2013), we identified additional markers associated with sensitivity to PF-309 in models of CRC and found that cells and tumors with an epithelial genotype (high *CLDN2*, *CDH1*, *RAB25*, *CLDN3*, *CDH17*) are more resistant and those with a mesenchymal genotype (high *CALD1*, *VIM*, *ANK2*, *ZEB1*) display sensitivity. Indeed, GEO tumors possess an epithelial genotype, requiring a higher concentration of PF-309 to achieve effect, while the SW480 tumors possess a mesenchymal genotype, requiring a lower concentration of PF-309 to achieve effect (Figures 5, 7, and 8). Going back and looking at the *in vitro* data, we see that two lines where the absence of *ABCB1* expression did not predict PF-309 activity, HT29, and NCI-H508, resistance may be explained by an epithelial genotype (Figures 1 and 8). Interestingly, in the panel of CRC cell lines, we also found that *ABCB1* expression significantly correlated with the epithelial genotype (*p* < 0.001, Figure 8A). This association may be an area of research worth further investigation.

The identification of patient populations expected to derive the greatest benefit from new therapies is a critical component in the development of molecularly targeted agents (Banck and Grothey, 2009; Prenen et al., 2010; Siddiqui and Piperdi, 2010; Heakal et al., 2011; Shaw et al., 2011). However, we must be careful not to ignore the need for adequate delivery of drug to tumors and the role that MDR proteins can play in this. The efficacy of compounds that are substrates for P-gp are not always highly affected by the presence of P-gp (Jang et al., 2001a), and we speculate that efficacy of PF-309 may be more greatly affected by the presence of P-gp due to it being a poorly permeable compound, although this is an area that requires further study (Feng et al., 2008; Sugano et al., 2010). Furthermore, total plasma concentrations of PF-309 were around 10–100 nM at the *C*_max_ (1 h) and 1–10 nM at the trough (6–8 h), which may not be sufficient to saturate P-gp efflux to allow greater intracellular accumulation.

Significant effort has been made in the development of agents to reverse or inhibit the effects of efflux transporters in order to restore or improve sensitivity of known substrates (Robert, 1999; Gottesman et al., 2002; Leonard et al., 2003). However, these efforts have thus far not been highly successful (Ries and Dicato, 1991; Dalton et al., 1995; Weinlander et al., 1997; Warner et al., 1998). We feel that for compounds such as PF-309, strong transporter substrates with poor permeability characteristics, a reasonable alternative to P-gp reversal may be to use patient P-gp status, in conjunction with other molecular classifiers of resistance (i.e., our epithelial/mesenchymal classifier for PF-309), to negatively select patients for therapy. Given the data presented here and in our companion paper (Pitts et al., 2013) we do not feel that we can predict which may play a larger role in resistance, P-gp or the epithelial phenotype. We feel that they must be evaluated together. Tumors that express high P-gp and an epithelial phenotype should be excluded from therapy and those with low/no P-gp and a mesenchymal phenotype should be included. In the other groups (High P-gp + M-type, Low P-gp + E-type) the data is less clear; for instance, it appears that if an m-phenotype exists cells/tumors are inherently more sensitive to PF-309 and therefore P-gp may not be as large of a barrier because extremely low concentrations are capable of inhibiting growth. Although we suggest that negative patient selection may be warranted for PF-309, we also recognize that upregulation of MDR transporters can occur following the initiation of therapy, which would lead to the eventual resistance to PF-309. Another alternative would be to investigate the use of PF-309 in cancer types, such as lung carcinomas, where PAKs play a role in disease progression, but transporter expression is generally low (Linn and Giaccone, 1995; Ong et al., 2011).

## Conflict of Interest Statement

Erica Lynn Bradshaw-Pierce, Leslie Nguyen, Mark West, Nathan V. Lee, Julie L. C. Kan, and Brion William Murray are all either current or former employees of Pfizer, Inc. S. Gail Eckhardt has received Research Funding, Fellowship Funding, and Honoraria from Pfizer.

## Footnotes

^1^(https://cabig.nci.nih.gov/)
